# Ownership and Use of Commercial Physical Activity Trackers Among Finnish Adolescents: Cross-Sectional Study

**DOI:** 10.2196/mhealth.6940

**Published:** 2017-05-04

**Authors:** Kwok Ng, Jorma Tynjälä, Sami Kokko

**Affiliations:** ^1^ Research Centre for Health Promotion Faculty of Sport and Health Sciences University of Jyvaskyla Jyväskylä Finland

**Keywords:** social determinants of health, mobile phone, health promotion, disabled children, physical activity, adolescent

## Abstract

**Background:**

Mobile phone apps for monitoring and promoting physical activity (PA) are extremely popular among adults. Devices, such as heart rate monitors or sports watches (HRMs/SWs) that work with these apps are at sufficiently low costs to be available through the commercial markets. Studies have reported an increase in PA levels among adults with devices; however, it is unknown whether the phenomena are similar during early adolescence. At a time when adolescents start to develop their own sense of independence and build friendship, the ease of smartphone availability in developed countries needs to be investigated in important health promoting behaviors such as PA.

**Objective:**

The objective of this study was to investigate the ownership and usage of PA trackers (apps and HRM/SW) among adolescents in a national representative sample and to examine the association between use of devices and PA levels.

**Methods:**

The Finnish school-aged physical activity (SPA) study consisted of 4575 adolescents, aged 11-, 13-, and 15-years, who took part in a web-based questionnaire during school time about PA behaviors between April and May 2016. Binary logistic regression analyses were used to test the associations between moderate to vigorous physical activity (MVPA) and devices, after controlling for gender, age, disability, and family affluence.

**Results:**

PA tracking devices have been categorized into two types, which are accessible to adolescents: (1) apps and (2) HRM/SW. Half the adolescents (2351/4467; 52.63%) own apps for monitoring PA, yet 16.12% (720/4467) report using apps. Fewer adolescents (782/4413; 17.72%) own HRM/SW and 9.25% (408/4413) use HRM/SW. In this study, users of HRM/SW were 2.09 times (95% CI 1.64-2.67), whereas users of apps were 1.4 times (95% CI 1.15-1.74) more likely to meet PA recommendations of daily MVPA for at least 60 min compared with adolescents without HRM/SW or without apps.

**Conclusions:**

To our knowledge, this is the first study that describes the situation in Finland with adolescents using PA trackers and its association with PA levels. Implications of the use of apps and HRM/SW by adolescents are discussed.

## Introduction

According to national statistics in Finland, by the age of 16 years, all Finnish adolescents have used the Internet in the past 3 months, 89% use the Internet several times a day, and 96% access the Internet via a mobile phone [1]. Less is known about younger adolescents aged between 11 and 15 years. These younger adolescents fall into the category of “digital natives.” It represents a generation that has lived in an age where the Internet has always been around [2]. Yet, there is an expectation that adolescents use technology in everyday life while at their homes, schools, and during leisure time, it is surprising how little there is research in the area of digital devices and apps for physical activity (PA).

Adolescence is an important period in a person’s life. In addition to the biological changes to the structure of the body, in developed countries, many young adolescents start to reduce their reliance on their family and start to become independent and engage in social activities [3]. It is also a time of life where PA behaviors start to decline, or if sustained, seem to continue into adulthood [4]. Therefore, much attention has been directed to public health officials to act and provide appropriate contexts to reduce the drop off in PA levels [5,6].

The World Health Organization has provided a set of PA recommendations for people to meet, in order to benefit their health. These recommendations include taking part in at least 60 min of moderate to vigorous physical activities every day for children aged between 5 and 17 years [7]. In Finland, the levels of PA have been moderate (boys: 29.7%; girls: 17.7%) when compared with other European countries (boys: 23.4%; girls: 13.9%) [8]. For example, the proportion of 11-year old Finnish adolescents who meet the international PA recommendations [7] is the highest in Europe and North America [9]. However, the overall grade for various PA determinants in the Global matrix was a C, which is comparable with the European average [6]. Differences have been reported by gender, whereby more boys (23.4%) meet the PA recommendations than girls (13.9%) [8]. There is also a decline in PA levels as age increases from 49% in primary school to 18% in lower secondary school that meet the PA recommendations [10]. In addition, despite Finnish adolescents experience the least amount of inequality to other countries in Europe [11], there is an association between higher financial wealth status and more days of reported PA [12].

Commercial PA trackers are more popular than ever. The multifunctional purposes that these trackers possess include data about personal health and fitness management, which has made them into one of the top desirable digital consumer products [13]. Competition in the design and production is intense, with regular consumer and scientifically led tests between products [14]. Recent test results of commercial products have demonstrated some level of acceptability as well as room for improvement when reporting overall PA levels [15-18]. These devices can come in the form of a wristwatch, a mobile phone app as well as a noninvasive piece of equipment, such as a strap that monitors the heart rate [19]. There is also a much debate about the potential sensors have on personalized health [20]. Self-quantification is a growing area as it has the potential to inform individuals [21]. In addition, it has the capability to share information publicly or anonymously among peers. However, many of these studies neglect investigations from adolescents. Of studies that have investigated social media use by adolescents, many have been on risk behaviors [22], whereas a few actually explore the use in a sporting context [23].

Mobile phone apps are tools on the users’ smartphone, whereas heart rate monitors or smart watches (HRMs/SWs) can operate independently from the phone. There are different levels of specifications from the hardware devices of HRM/SW. App user experiences are often richer than hardware devices, although there can be some overlap. For example, Polar HRM/SW has the capability to be linked with a mobile phone app, consequently the user has access to more data that the HRM/SW itself.

The majority of studies that used apps as a technique to positively change health behaviors were successful [24]. These findings are useful in the development of behavioral theories that connect personalized data with tracked behaviors, including PA, onto the Internet. People with health conditions or functional limitations, which are severe enough to cause a disability, have also benefited from apps [25,26]. Moreover, the apps are a popular tool used by adults in the mainstream population [27]. From this, there has been an assumption that PA trackers can support PA behaviors [28]. However, what many studies have done is to report the reliability of the devices [14,15,18], the benefits from wearing devices [28], as well as devices failing to have an effect on weight loss [29]. Intervention studies on adolescents have been rare [30], although the use of devices seems promising in its effect in reduction of screen-time, the long-term impact of such results are still unknown [31]. Ridgers and colleagues [30] found three interventions and two feasibility studies up until August 2016. In one of the intervention studies, Fitbit Ones were used a tool to promote PA to children with leukemia. However, despite overall happiness to use the PA tracker, there were no significant increases in step-count [32]. In comparison to studies with adults, the studies do not inform us whether the low number of studies reported is because of low usage among adolescents. To address this concern, the purpose of this study was to investigate the prevalence of adolescents who report on ownership and use PA measuring devices. In addition, this study examines the strength of association between ownership and use of devices and meeting adolescents PA recommendations.

## Methods

### Recruitment

The Finnish school-aged physical activity (SPA) study is a national representative cross-sectional study of many determinants of PA among adolescents aged between 11 and 15 years. Participants were selected at random, based on a stratified sampling method set by regional stratification in the capital, southern, central, and northern Finland. The class was the primary sampling unit through probability proportion size. In total, 109 Finnish-speaking schools and 65 Swedish-speaking schools participated. The response rates were 61% from the Finnish speaking schools, and 58% from the Swedish speaking schools [33]. Through proportional to size of school, the units were selected and a reserve list created to be used if necessary. The questionnaire was completed online in a computer class for a maximum of 60 min with an instructed teacher who presided over the data collection. The questionnaire was completed anonymously, voluntarily, and the study has been approved by the Institutional Ethical Review Board Committee.

### Measures

Adolescents were asked to report their gender (boy or girl). They were also asked to report their month and year of birth. The ages were then grouped into 11-, 13-, and 15-year old age groups based on their age closest to an age group category at the time of completing the survey.

#### PA Trackers

Ownership and usage of PA tracking devices were measured through two items. Both items began with the following opening question, “Do you have any of the following PA measuring devices...,” and were followed by (1) app and (2) HRM/SW. The response categories of these measures were “I do not have,” “Yes, but do not use it actively,” and “Yes and use it actively.” The wording used in the items were selected after extracting marketing materials of leading commercial PA measuring device companies in the Finnish language. The items were later translated into English for reporting.

#### Physical Activity Levels

Overall PA was measured through a single-item self-reported measure. As part of this measure, there was a pretext description of moderate to vigorous physical activity (MVPA). In addition, some example activities typical for adolescents to do were stated so that they could understand what to include in their recalled response [34]. Following this description, the respondents were asked, “Over the past 7 days, on how many days were you physically active for a total of at least 60 min per day?” Only one response was allowed that ranged from 0 to 7 days. For the study’s purposes, the mean number of days was used to examine the differences in the average per group. In addition, a dichotomous cutoff based on the international PA recommendations [7] (7 days) versus not meeting the recommendations (between 0 and 6 days) was used for inferential statistics. The question has been widely used with acceptable validity and reliability in adolescent surveys [35,36]. The question has a significant validation correlation coefficient of .40 from adolescents in clinical settings [34] and is used widely in national and international adolescent health studies [37]. In Finland, the ICC values from a 2-week test-retest were between .7 and .8 [35]. Although there is criticism on the use of subjective reporting of PA, the use of self-reported PA in surveillance studies has been considered an approved method [38].

#### Disability

Data were disaggregated by disability through items used in the WHO Model Disability Survey [39]. Individuals were asked to report their severity of functional difficulties, including seeing, speaking, hearing, moving, breathing, and remembering or concentrating. These items were modified from the WHO Model Disability Survey to disaggregate by disability with a 5-point scale to indicate severity [40].

Severity was determined by functional difficulties that were of 1 (no difficulty), 2 (mild difficulty and did not affect participation), 3 (moderate difficulty and affects participation), 4 (severe difficult and affects participation), and 5 (complete difficulty and affects participation). To meet the criteria of having a disability, adolescents had to have reported at least one functional difficulty with severity of 3 or higher. In other words, the individual felt the difficulty affected their participation.

#### Family Affluence

The Family Affluence Scale (FAS) III is an international measure of social economic status of adolescents [41]. It comprises of six items related to family cars, holidays, computers, rooms, bathrooms, and dishwasher at home. An internationally established formula [41] was used to define three groups of family affluence: low, middle, and high [42].

### Statistical Analysis

Study characteristics, such as age, gender, disability, and FAS were described through ownership and usage of mobile phone apps or HRMs/SWs, and were tested by chi-square test of independence (*P*<.05). Mean number of PA days were reported by each participant characteristic. Binary logistic regression analyst was conducted on the overall sample through dichotomized groups of participants that reported 0-6 days of MVPA and 7 days of MVPA. Adjusted odds ratios (OR) and 95% CIs were reported to indicate the likelihood of daily MVPA. To report positive odds, girls, who were aged 15 years, with low FAS and disability were reference groups. Furthermore, adolescents who had no PA trackers were also the reference groups. SPSS version 24.0 for Windows (SPSS Inc) was used for the analyses.

## Results

### Descriptive Statistics

In this study, half (2434/4575; 53.20%) of the 4575 adolescents (mean age 13.8 years, SD 1.64) were girls. Just over a quarter (1262/4575; 27.58%) of adolescents reported daily MVPA. Adolescents identified to have disabilities had indicated that their difficulty severity level was between “moderate” to “complete.” Whereas, responses of severity levels of “mild” and “none” were grouped into adolescents without disabilities. On the basis of this definition, one in six (672/4568; 14.71%) adolescents reported to have disabilities. Family affluence was more skewed toward middle (2445/4575; 53.44%) and high (1650/4575; 36.07%) FAS, hence adolescents with low FAS (480/4575; 10.49%) were in the minority (Table 1).

**Table 1:** Sample characteristics of mean days of moderate to vigorous physical activity (MVPA) with 95% CI and proportion of meeting physical activity (PA) recommendations.

Characteristics		n	Mean (SD^a^)	95% CI	% MVPA^b^
**Gender**					
	Girls	2434	4.71 (1.82)	4.64-4.79	22.51
	Boys	2141	5.05 (1.90)	4.96-5.12	33.35
**Age groups (in years)**					
	11	1584	5.41 (1.77)	5.41-5.32	40.15
	13	1594	4.90 (1.77)	4.81-4.99	25.72
	15	1397	4.22 (1.89)	4.12-4.33	15.46
**Disability**					
	With	672	4.44 (2.04)	4.28-4.59	23.21
	Without	3896	4.94 (1.82)	4.88-5.00	28.29
**Family affluence**					
	Low	480	4.64 (1.98)	4.46-4.82	25.63
	Middle	2445	4.77 (1.90)	4.69-4.84	25.44
	High	1650	5.07 (1.80)	4.99-5.16	31.33
**HRM/SW^c^**					
	No	3223	4.72 (1.90)	4.65-4.78	25.04
	Unused	782	5.06 (1.74)	4.94-5.18	28.77
	Uses	408	5.70 (1.59)	5.54-5.86	44.12
**Apps**					
	No	2116	4.65 (2.00)	4.57-4.73	25.09
	Unused	1631	4.93 (1.78)	4.84-5.02	26.92
	Uses	720	5.37 (1.67)	5.24-5.49	35.70

^a^SD: standard deviation.

^b^%MVPA: percentage that meet international physical activity recommendations for adolescents.

^c^HRM/SW: heart rate monitor or sport watch.

Of the adolescents who report ownership but not active use of apps, only (104/1579) 6.59% reported to use HRM/SW. Under one-third of app users (207/694; 29.8%) reported to use HRM/SW. Just over half (207/391; 52.9%) of HRM/SW users reported that they were users of apps too.

### Mobile Phone Apps

More girls (1307/2351; 55.59%) reported that they have apps that measure PA than boys (1044/2351; 44.41%, *P*=.003). In addition, as the age of adolescents increased, the more apps owned to measure PA (*P*<.001). Among all the adolescents, one in six (720/4467; 16.12%) reported that they were using the apps to measure PA (Table 2).

Ownership of apps and meeting the PA recommendations was not always positive (OR =1.11, 95% CI 0.94-1.30). Apps usage was positively associated with the PA recommendations (OR 1.41, 95% CI 1.15-1.74) than adolescents without apps to track PA, after controlling for gender, age, disability, and FASIII.

### Hear Rate Monitors and Sport Watches

More boys (590/2051; 28.77%) reported to own or use HRM/SW than girls 600/2362; 25.40%). One in ten (408/4413; 9.25%) adolescents reported to use HRM/SW. Almost half (325/694; 46.8%) of the active users of mobile phone apps that measure PA reported to have HRM/SW, although only 29.8% (207/694) were also using HRM/SW. There were more ownership and usage of HRM/SW in adolescents with higher family affluence groups (Table 3).

There were stronger associations with HRM/SW and PA. The association between owning HRM/SW and meeting the PA recommendations was not necessarily positive all the time (OR 1.18, 95% CI 0.97-1.42). Adolescents who owned and used HRM/SW were 2.09 times (95% CI 1.64-2.67) more likely to meet the PA recommendations than adolescents without HRM/SW, after controlling for gender, age, disability, and FAS III.

**Table 2:** Sample characteristics of users of smartphone apps and chi-square test of independence.

Characteristics		Smartphone apps				*P* value
		Total, n	None (%)	Yes and unused (%)	Yes and used (%)	
**Gender**						.01
	Boys	2077	1033 (49.74)	717 (34.52)	327 (15.74)	
	Girls	2390	1083 (45.31)	914 (38.24)	393 (16.44)	
**Age groups (in years)**						<.001
	11	1537	788 (51.27)	490 (31.88)	259 (16.85)	
	13	1560	721 (46.21)	591 (37.88)	248 (15.90)	
	15	1370	607 (44.31)	550 (40.15)	248 (18.10)	
**Disability**						.75
	With	652	313 (48.0)	230 (35.3)	109 (16.7)	
	Without	3810	1802 (47.29)	1400 (36.75)	608 (15.96)	
**Family affluence**						<.001
	Low	474	291 (61.4)	123 (25.9)	60 (12.7)	
	Middle	2381	1199 (50.35)	856 (35.95)	326 (13.69)	
	High	1612	626 (38.83)	652 (40.45)	334 (20.72)	
**60 min MVPA^a^**						<.001
	Not daily	3240	1585 (48.92)	1192 (36.79)	463 (14.29)	
	Daily	1227	531 (43.28)	439 (35.78)	257 (20.95)	
Total		4467	2116 (47.37)	1631 (36.51)	720 (16.12)	

^a^MVPA: moderate to vigorous physical activity.

**Table 3:** Sample characteristics for users of heart rate monitors or smart watches and chi-square test of independence.

Characteristics		Heart rate monitor or sports watch				*P* value
		Total, n	None (%)	Yes unused (%)	Yes and used (%)	
**Gender**						.04
	Boys	2051	1461 (71.23)	387 (18.87)	203 (9.90)	
	Girls	2362	1762 (74.60)	395 (16.72)	205 (8.68)	
**Age groups (in years)**						.07
	11	1517	1140 (75.15)	235 (15.49)	142 (9.36)	
	13	1542	1113 (72.18)	284 (18.42)	145 (9.40)	
	15	1354	970 (71.64)	263 (19.42)	121 (8.94)	
**Disability**						.52
	With	637	461 (72.4)	122 (19.2)	54 (8.5)	
	Without	3770	2761 (73.24)	659 (17.48)	350 (9.28)	
**Family affluence**						<.001
	Low	466	383 (82.2)	62 (13.3)	21 (4.5)	
	Middle	2357	1814 (76.96)	357 (15.15)	186 (7.89)	
	High	1590	1026 (64.53)	363 (22.83)	201 (12.64)	
**60 min MVPA^a^**						<.001
	Not daily	3201	2416 (75.48)	557 (17.40)	228 (7.12)	
	Daily	1212	807 (66.58)	225 (18.56)	180 (14.85)	
Total		4413	3233 (73.26)	782 (17.72)	408 (9.25)	

^a^MVPA: moderate to vigorous physical activity.

## Discussion

### Principal Findings

The findings of this study suggest that over half of adolescents own mobile phone apps that can function as PA trackers, however, one in six reported to actually use the apps. In addition, at the time of the study, a quarter of adolescents own HRM/SW yet, one in ten used HRM/SW.

### Phone Apps Are Popular for PA Tracking

In light of the official statistics in Finland, by the time adolescents reach the age of 16 years, almost all (96%) of adolescents can access the Internet via mobile phones [1]. This high usage that was expected with the main reason has attributed to the absence of landlines in Finland since the 1990s and has encouraged mobile phones use among adolescents [43]. The findings from this study reveal the majority of adolescents (2351/4467; 52.63%) have sufficient interest in PA behaviors to have PA tracking apps on their phones.

A poll found that downloading apps were the third most popular function (82%), behind taking photos and messaging friends in adolescents aged between 13 and 16 years from the United Kingdom [44]. Data on electronic media usage reveals that three quarters of Finnish adolescents aged between 10 and 14 years use mobile phones and social media takes up the majority of their free time [45]. Other activities such as social media, surfing the Internet, playing games, watching videos, reading, and listening to music can take up the most amount of time on electronic media. Not all smart phones have, by default, PA tracking apps. However, they can include hardware components, such as accelerometers and GPS, which are key functions to track PA. To enable these hardware components, users often need to be aware of the functions, activate the apps, and feel the desire to install the apps.

Previous research has shown PA as a socially desirable habit [46], although in the case of these adolescents, one in six reported to use the apps for the purposes of monitoring PA. The availability of these apps on the phones, and low usage highlights the context in which adolescents use the mobile phone as an organic part of everyday life [43]. App designers may need to target adolescents who carry their phone with them everywhere, by encouraging a fun way that is also purposeful to meet their daily electronic usage needs and at the same time for self-input PA data. Games like Pokémon Go seem to have a mechanism to achieve this. However, an update to this would be providing more support by tailoring their existing PA levels and providing feedback on habitual PA. This may spur on interest in the differences between simply owning an app and actual usage of apps.

There was a positive association between the usage of apps and meeting the current international PA recommendations. The apps connect with various sensors on the phone and translate the data from algorithms to the user interface [47]. Despite the varying levels of accuracy when the apps produce information [24,48], it is a starting point for quantifying the self in PA behaviors [21]. This type of self-quantification needs to be explored among digital natives since previous research has excluded responses by adolescents aged between 14 and 17 years [13].

The international PA recommendations are there to encourage children aged between 5 and 17 years to take part in daily MVPA [7]. According to the results from this study, the odds of active users of mobile phone apps to meet the recommendations were 1.4 compared with the reference group of nonowners of PA tracking apps. Apps have enormous potential to provide personal and immediate behavioral feedback. In control theory [49], feedback loops can direct behavior toward desirable patterns. The positive reinforcement by the feedback is more than self-quantification. Adolescents can use the apps and connect to other users [47]. The individual can share their results with their friends. This can encourage users to be more physically active, to demonstrate the recommendations are met, as well as bring up a point of discussion between users for ways to take part in enjoyable activities.

Apps that display information for personalized feedback can motivate individuals to be more physically active [50]. This is an important health promotion opportunity. One of the main ways to combat noncommunicable disease is to decrease physical inactivity. Along with the popular behavior theories to support behavioral change techniques [51], researchers may need to start thinking about how apps play a role in the advancement of such developments. The results from this study highlight the need for developers of apps and researchers who use apps for interventions to recognize that an app is not enough. Often one-size-fits-all in traditional health promoting interventions do not work [52]. Through changes in software, apps that read individual data can give more personalized feedback [30]. This is important to reach the populations that are physically inactive since, setting sights on an immediate change to daily MVPA may seem too off putting for them.

Compared with other intervention methods, one main advantage that apps have over other techniques are the tailoring mechanisms for each individual. Apps can be programmed to create easy-to-reach goals in the early stages of PA promotion, and then slowly progress toward the PA recommendations and beyond with the user in control. Therefore, it is recommended that there are apps developments that take into account segmentation in gender, age, dis/ability, and wealth.

### Heart Rate Monitors or Sports Watches as Specific Devices

Devices such as HRM/SW require extra user commitment such as maintenance, care, battery charging, turning on and off. Therefore, it was no surprising that users of HRM/SW had the largest ORs for meeting PA recommendations. Moreover, it is most likely that users of HRM/SW are early adopters of PA tracking devices as they have already invested a large amount of their time in PA and sports. The feedback loop, under control theory [49], from HRM/SW has more emphasis than in apps. Feedback from HRM/SW is readily available from a display, usually positioned on the wrist. A quick glance can modify motivation levels to take part in more activity so that the individual meets the daily targets. Alternatively, the user may be looking for confirmation that they have reached their target so that they can choose other activities, such as school work, socializing, arts, or just relaxing. These prompts are inviting for users who would want a device to help them meet the PA recommendations. However, motivational nudges to change behavior have the risk of polarization. Whereby individuals who do not meet the recommendations due to other difficulties, resist the feedback and find new gadgets distasteful. Feasibility studies from adolescents suggest that comfort, design, and feedback features are more important than the usability and effectiveness for increasing PA behaviors [30]. As with apps, interventions with HRM/SW may need a tailoring approach for effective health promotion in adolescents.

Today’s PA devices worn on the wrist have the capability to measure the number of steps taken during the day and can be compared with the recommended step count level that is equivalent to 60 min of MVPA [53,54]. Physical and health educators may want to start considering how to integrate this knowledge into their messages. Furthermore, should HRM/SW be proven to be effective in PA promotion, national agencies may want to consider the creation of subsides to assist purchasing of equipment, thus allowing pupils to learn about self-quantification in the classes. This development is only the beginning, as newer devices become more readily available, with more functions, more feedback preferences, and at lower costs [48].

### Subpopulation Differences in Ownership and Usage of PA Trackers

Tailoring of PA trackers may also need to consider the gender differences reported in this study. More girls reported that they had apps to measure PA than boys. Goal orientation toward PA tends to differ between boys and girls; for example, girls tend to take part in PA for enjoyment, whereas boys take part in PA for its competitive nature [55]. Another issue could be the changes in maturation between the genders during these ages. During early adolescents, studies have reported that girls have stronger digital literacy skills than boys [56]. Boys may know less about the use of apps on the mobile phones [57]. However, more boys who reported to have PA trackers reported to actually use the apps and HRM/SW. The mechanisms to use the trackers may be common only among boys who have an interest in the behavior. Consequently, this would indicate that the use of PA trackers were to support competitive motives through PA rather than to be used as an intervention tool to promote more PA behaviors.

There was an increase in the number of users of PA trackers as the cohorts increased with age. Traditionally, there have been substantial declines in PA levels to the users who are aged between 11 and 15 [12]. Many potential benefits from these designs are possible. At the age of 11 years, adolescents are usually active, requiring fewer interventions than 15-year-olds. Usually at the turn from young to mid adolescents, there is a need to fit in socially [3]. Technology use to aid social interactions is second nature for digital natives. Understanding this phenomenon by digital natives is an important consideration for health promoters as some traditional methods have low relevance for young adolescents.

It was no surprise that high family affluence was positively associated with PA trackers. Although the majority of apps are freely available to download from the mobile phone app stores, families must provide adolescents with phones that have sufficient capabilities for measuring PA. Moreover, the combination of peripheral devices (HRM/SW) can be quite an expensive acquisition for some families. Previous studies have reported the association with higher family affluence and increases in PA levels in adolescents [12], and it now extenuated with ownership of HRM/SW, which was also associated with more days of reported PA.

Adolescents without disabilities reported more days of MVPA than adolescents with disabilities. However, the differences were not strong enough to influence the statistical model. This concurs with previous studies from mainstream school populations whereby there were no significant differences in PA levels between adolescents with and without disabilities [58,59]. In this study, the differences among the various functional differences were not reported neither since, of all the different functional difficulty categories, only adolescents with moving difficulties were significantly less active than other adolescents [60]. The usage and ownership of PA trackers were also indifferent between adolescents with and without disabilities. These results can be used to inform health practitioners to use mobile health apps and other devices as part of targeted interventions for adolescents despite their health conditions.

### Study Limitations

The results of this study have some limitations. Data collected were from a cross-sectional study design. Therefore, the associations between devices and PA are not causal. In other words, the results should equally be read so that adolescents who are more interested in being physically active have devices, as adolescents have devices do so to taking part in more daily PA. Intervention and longitudinal studies might provide better insight into the causal links, behavioral techniques to increase PA levels, and commercialization of products to reinforce the sense of athletic identity. More studies that utilize longitudinal designs to investigate the relationships between PA trackers and its influences on PA are strongly recommended. PA levels were collected through self-report methods. There may be some over or under reporting bias; however, because all respondents used the same method, it has been possible to find the associations between PA trackers and PA levels. In addition, the items that measured PA trackers have not been tested for meeting minimum reliability and validity scores. The results of the studies were limited to an undefined use of apps or HRM/SW. Future studies may consider development of these items to help differentiate the types of users across activity trackers. Future studies may need to improve the items used to eliminate as much error as possible.

### Conclusions

This study has reported the ownership and usage of PA trackers among adolescents in Finland. It is an exciting time for researchers as digital natives use accessible tools for their own personal health. In this study, the appeal of PA trackers was evident, as over half of Finnish adolescents have mobile phone apps to track PA. The strongest association reported was among the one in ten adolescents that have HRM/SW and took part in daily PA to correspond with the current PA recommendations for health [7]. However, only 22.51% (548/2434) and 33.35% (714/2141) of girls and boys, respectively, reported to meet these recommendations. Other differences in age and wealth were observed, although not in disability. More understanding is needed on the mechanisms to build suitable apps and devices for adolescents as they live through bodily and supportive changes in the life, while promoting healthy behaviors. Apps and device development would benefit from understanding the individual differences in current PA levels and relative targets.

## Abbreviations

FAS: Family Affluence Scale III
HRM/SW: heart rate monitor or sports watch
MVPA: moderate to vigorous physical activities
OR: odds ratio
PA: physical activities
SPA: school-aged physical activity
WHO: World Health Organization
